# Aetiologies of Central Nervous System infections in adults in Kathmandu, Nepal: A prospective hospital-based study

**DOI:** 10.1038/srep02382

**Published:** 2013-08-08

**Authors:** Abhishek Giri, Amit Arjyal, Samir Koirala, Abhilasha Karkey, Sabina Dongol, Sudeep Dhoj Thapa, Olita Shilpakar, Rishav Shrestha, Le van Tan, Bkrong Nguyen Thi Thuy Chinh, Radheshyam Krishna K. C., Kamal Raj Pathak, Mila Shakya, Jeremy Farrar, H. Rogier Van Doorn, Buddha Basnyat

**Affiliations:** 1 Oxford University Clinical Research Unit- Patan Hospital, Nepal; 2 Oxford University Clinical Research Unit- Vietnam, The Hospital for tropical disease, Wellcome trust Major Overseas programme, Ho Chi Minh City, Vietnam; 3 Centre for Tropical Medicine, University of Oxford, Oxford, United Kingdom; 4 Patan Academy of Health Sciences, Patan Hospital, Nepal

## Abstract

We conducted a prospective hospital based study from February 2009-April 2011 to identify the possible pathogens of central nervous system (CNS) infections in adults admitted to a tertiary referral hospital (Patan Hospital) in Kathmandu, Nepal. The pathogens of CNS infections were confirmed in cerebrospinal fluid (CSF) using molecular diagnostics, culture (bacteria) and serology. 87 patients were recruited for the study and the etiological diagnosis was established in 38% (n = 33). The bacterial pathogens identified were *Neisseria meningitidis* (n = 6); *Streptococcus pneumoniae* (n = 5) and *Staphylococcus aureus* (n = 2) in 13/87(14%). Enteroviruses were found in 12/87 (13%); Herpes Simplex virus (HSV) in 2/87(2%). IgM against Japanese encephalitis virus (JEV) was detected in the CSF of 11/73 (15%) tested samples. This is the first prospective molecular and serology based CSF analysis in adults with CNS infections in Kathmandu, Nepal. JEV and enteroviruses were the most commonly detected pathogens in this setting.

Globally central nervous system infections limited to the meninges (meningitis) or with brain parenchyma involvement (encephalitis) are common causes of hospital admissions. The pathogens responsible for these infections may be bacteria, viruses, fungi or parasites. The incidence and etiology of CNS infections vary in time, by geographic region, with age, co-morbidities and vaccination policies. It is important to document the causative agents for these infections to improve public health measures and clinical practice.

The most common bacterial CNS pathogens both in adults and children are *Streptococcus pneumoniae* and *Neisseria meningitidis*^1^. *Haemophilus influenzae* type b causes bacterial meningitis in children, but its incidence has decreased in areas where an effective vaccine has been incorporated into national programme^2^. *Streptococcus suis*, a zoonotic pathogen from pigs, is one of the most important causes of meningitis in adults in Southeast Asia^3^. Tubercular meningitis (TBM), a severe form of extra pulmonary tuberculosis, is presumed to be common due to high prevalence of tuberculosis in Southeast Asia^4^. However, there are no definitive estimates on incidence of TBM or any other bacterial meningitis in Nepal.

Aseptic meningitis and encephalitis are the other important types of CNS infections. Enteroviruses are the most common organisms known to cause aseptic meningitis^5^. However, a multitude of pathogens may be associated with encephalitis varying by geographic region, age group of patients, and time. The majority of these pathogens remain unidentified in routine clinical diagnostics. Herpes simplex virus (HSV) was found to be the predominant cause of acute encephalitis in studies from the western world^6^,^7^,^8^. Studies done in Southeast Asia and the Western Pacific regions have shown Japanese encephalitis virus (JEV) as the leading cause of acute encephalitis especially in children and young adults^9^. In other countries of Southeast Asia (Bangladesh and India) Nipah virus and enteroviruses remain a major public health concern^10^,^11^. However, no study has reported these viruses in Nepal.

The epidemiological data related to the etiology of CNS infections in Nepal is limited. JEV is endemic in the Terai (southern) region and in few hilly districts of Nepal [Figure 1]. JEV has been identified as the most common cause for acute encephalitis syndrome (AES) in Nepal. WHO surveillance studies in Nepal have shown JEV as an important cause of AES especially in children <15 years of age, but 25–40% of JEV confirmed encephalitis have been reported in age group >15 years^12^. However, these studies have relied on JEV confirmation primarily by single serum serology in the context of an encephalitic illness which may potentially overestimate the disease burden in an endemic setting. Previous hospital-based studies in Nepalese children have shown *S. pneumoniae* and *H. influenzae* type b as the most common bacterial pathogens and JEV as the major viral pathogen for CNS infections^13^,^14^,^15^,^16^. However, the etiology of encephalitis apart from JEV and the pathogens involved in CNS infections in adults remain unknown. The previous studies have been done in children using bacterial culture or serum serological assays and no study report using molecular diagnostic methods has been reported on CNS infections from Nepal. Hence to further explore the aetiology of CNS infections in Nepal, we conducted a hospital based study using molecular diagnostic techniques [real-time (reverse transcription [RT]) PCR] and CSF serology.

## Results

In total, 168 patients were assessed for their eligibility in the study. 87 patients were included in the analysis. CSF samples from these 87 patients were available for bacterial and viral (RT) PCR. Only 73 CSF samples were sufficient for serology and 68 CSF culture reports were available.[Figure 2].

The median age of the study patients was 30 years (IQR: 22–54). 57% (n = 50) were male. Our hospital being a tertiary referral center, 55% of the study patients (n = 48) received either treatment or were referrals from other hospitals. The median duration of illness on presentation was 7 days (IQR: 8–14 days). The clinical syndrome was indistinguishable among different infections except for the differences in CSF picture in bacterial infections. Patients infected with a bacterial pathogen had low glucose (p = 0.02), high protein (p = 0.03), and CSF pleiocytosis (p = 0.01) as compared to viral infections. Bacterial infections (compared to viral infections) also presented more in younger adults either alone (P = 0.02) or as a co-infection with other viruses (p = 0.02). No significant difference in clinical presentation was noted between enteroviruses and JEV. The demographic, laboratory and clinical features of the study patients are displayed and compared in [Table 1] & [Table 3].

A total of 38 CNS pathogens were identified in 33 patients (38%). Evidence of bacterial infection was found in 13 patients (14%), including *N. meningitidis* in 6 patients (6.8%) and *S. pneumoniae* in 5 patients (5.7%). *S. aureus* was identified from CSF culture in 2 patients out of 68 culture reports available [2/68, 3%]. 14 patients had samples that were positive for viral (RT-) PCR: enteroviruses (n = 12, 13%) and HSV (n = 2, 2%). None of the enteroviruses were found to be EV (71). Dual infections with enteroviruses were found in 3 patients. Serology for JEV was positive in 11 out of 73 (15%) samples tested among which were 3 dual infections. Serology test results for anti dengue virus IgM were negative for all the tested CSF samples.

Evidence of viral co-infection with a bacterial pathogen was found in CSF samples of 4 patients (*S. pneumoniae* with EV or JEV in 2 patients; *N. meningitidis* with JEV in 1 patient and *S. aureus* with enteroviruses in 1 patient). One patient had a dual infection with 2 viruses (JEV and enteroviruses). The results of (RT-) PCR, serology and culture of the study patients are displayed in [Table 2].

## Discussion

CNS infections impose a substantial disease burden and diagnostic challenge. The true estimates on these infections are difficult to ascertain especially in a resource-limited country like Nepal. Previous hospital based studies done in Nepal have been restricted to children and no study using a combination of classic and molecular diagnostic techniques on CSF has been reported. Nearly one third of the acute encephalitis syndromes (AES) are presumed to be due to JEV in Nepal^12^. For the rest, etiology is unknown. The incidence rate of acute encephalitis syndrome is estimated to be nearly 12 times higher in Nepal (5.23/100,000) than in neighboring India (0.42/100,000)^10^. Besides JEV, encephalitis outbreaks related to enteroviruses, Nipah virus, and Chikunguniya virus have been frequently reported from the neighboring regions^10^,^17^. These viruses have not yet been reported in Nepal. Due to similarities in topography and frequent movement of people to either side of these regions there is a possibility of these viruses causing similar outbreaks within the Nepalese territory. Our study attempts to explore these different pathogens known to cause CNS infections within our region. Our analysis reveals that enteroviruses and JEV are the most common causes of CNS infections in adult hospitalized patients in Kathmandu, Nepal. To our knowledge, this is the first report of CNS infections caused by enteroviruses in adults from Nepal.

Enteroviruses (EV) are well-established causes of aseptic meningitis/encephalitis in infants, young children and to a lesser extent in adults^5^. Outbreaks of enteroviral encephalitis in children have been reported from neighboring countries India^18^,^19^ and China^20^. Since 1997, there have been reports of large outbreaks of EV71 associated hand, foot and mouth disease among children from the Asia pacific region^21^,^22^. Although these outbreaks affected only children, we tested for EV71 in our patients but none were detected in our samples. Studies done outside Southeast Asia have reported enteroviruses as a common cause of CNS infections in adults^23^,^24^. However, only few studies from Southeast Asia have reported enteroviruses as an adult CNS pathogen. A provincial hospital based study done in Vietnam found enteroviruses in CSF by real-time RT-PCR in 10% of children and in 8% of adults with a CNS infection^25^. A recent hospital-based study in central India reported 11% of the adult patients with CNS infection had enteroviruses^26^. In our study, 13% (n = 12) of the patients with CNS infection had enteroviruses.

*S. pneumoniae (n = 5, 5.7%)* and *N. meningitidis (n = 6, 6.8%)* were the most common bacterial pathogens detected in 11/87 CSF samples tested by (RT-) PCR. These findings are similar to the other study reports on bacterial meningitis elsewhere from our region^27^,^28^. *S. aureus* was identified in CSF culture in 2 patients out of total 68 culture reports available. No evidence of bacterial growth was observed in remaining 66 culture reports. This could be due to prior antibiotic treatment received before hospital admission as 55% (n = 48) patients in the study received some treatment or were referrals to our hospital. *S. suis,* a zoonotic pathogen from pigs, has been frequently reported in adults in studies done in Southeast Asia^3^ but none were detected in our study. Tubercular meningitis (TBM) is a common diagnosis in our hospital, but the diagnosis relies entirely on the judgment of treating physician based on history, clinical features, CSF findings and supportive investigations (chest X-ray and sputum acid fast bacilli). Six out of 87 enrolled patients had a final clinical diagnosis of TBM with no confirmatory laboratory tests done. Interestingly, 2 enteroviruses and 1 HSV were found in these patients' CSF.

**Figure 1:**
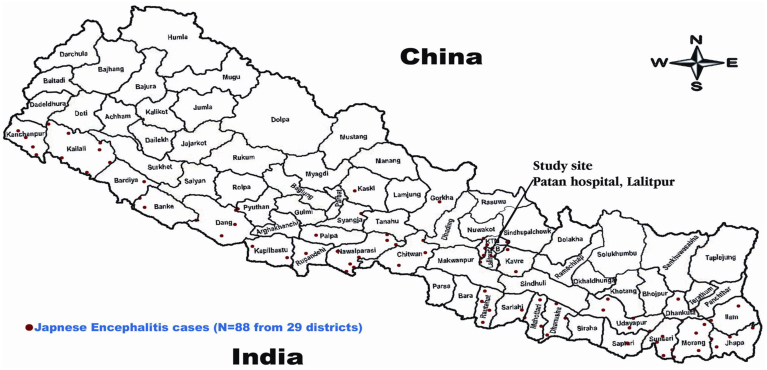
Geographical Map of Nepal showing distribution of Japanese encephalitis virus (JEV) for 2010/2011. Japanese Encephalitis cases (n = 88) from 29 districts are represented by (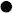). Study site -Patan Hospital, Lalitpur. Terai - southern regions adjacent to India. Source- Ministry of Health and population, Department of Health Services of Nepal, Annual Health Report 2067–2068(2010/2011).

**Figure 2:**
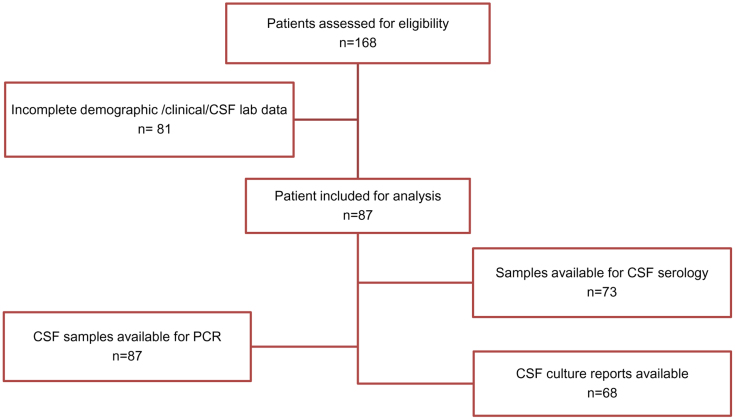
Flow diagram for total patients included in the study.

During the period of 1978 to 2003, nearly 26,700 suspected JEV infections with 5,400 deaths were reported in Nepal^29^,^30^. JEV is endemic in the Terai (southern) region of Nepal [Figure 1] and in the densely populated Kathmandu valley^13^,^15^,^31^. JEV rarely infects adults living in endemic regions presumably because of exposure to virus during childhood and subsequent immunity^32^. A provincial hospital based study from Vietnam found anti JEV IgM in CSF of 33% of children and in only 5% of adults with an encephalitic syndrome^28^. However, there have been reports of an age shift in JEV- infected patients from children to adults in India^33^. WHO surveillance studies in Nepal between 2009 and 2010 (during our study period) report 326 laboratory confirmed JEV out of which 102 cases (31%) were seen in age groups >15 years^12^. However, these estimates have been based on single serum serology in patients with an encephalitis illness which may potentially overestimate the disease burden in an endemic setting. In our study, out of 73 tested samples, 11 samples (15%) were positive for anti JEV IgM in the CSF. In previous studies done in Nepal^13^,^15^, JEV was confirmed based on serum serology (anti JEV IgM) and only a few diagnoses in these studies were confirmed using CSF based anti JEV IgM. In our study, we determined the CSF anti JEV IgM which has a higher diagnostic accuracy than one point serum serology^34^.

Surveillance for AES/JEV is a part of public health program in Nepal. Vaccinations for Japanese encephalitis and *H. influenzae* type b have been incorporated in the national immunization program due to a high estimated burden of these diseases especially in children. A pentavalent vaccine for diphtheria, pertussis, tetanus, hepatitis B and *H. influenzae* type b is given in 3 doses to children at 6, 10 and 14 weeks of age. A single dose of SA 14-14-2, live attenuated vaccine, is used for JEV in children between 12–23 months. Study reports from the western regions of Nepal have shown SA 14-14-2 to be highly efficacious and safe in prevention of Japanese encephalitis^35^,^36^,^37^. During 2006 to 2009, JEV vaccination campaigns were conducted in 23 endemic districts of Nepal. Vaccination campaigns targeted different age groups in various districts. In 11 districts (moderate risk) children 1–15 years of age were vaccinated and in 12 districts (4 high risk and 8 moderate risk) all person aged >1 year were vaccinated. A study report based on these mass vaccination campaigns has reported significant decrease in total number of AES and JEV confirmed encephalitis from these districts following vaccination. The high-risk districts where adults were vaccinated showed a higher decline in JEV than in the districts where only children (1–15 years) were vaccinated^38^. This study has emphasized the importance of JEV vaccination in reducing the disease incidence and also on the possible role of adult vaccination to reduce disease transmission in an endemic setting. However, a few important factors which were not considered during this study might have lead to inaccurate estimation of disease incidence rate (pre and post vaccination) and vaccine efficacy. These include- lack of confirmatory tests for JEV diagnosis, the possibility of other pathogens causing AES and the impact of simultaneous public awareness campaign and anti-mosquito programmes disrupting the JEV transmission cycle during these years. Hence there is a need for further studies to explore the other possible pathogens and to use specific confirmatory test for more accurate estimation of disease burden which might be very helpful in implementing cost-effective vaccination policies.

Our study had several important limitations. The study took place at an urban referral hospital over a limited time period. A large number of patients (n = 81) were not included in the analysis hence we could not ascertain the complete variety and the seasonality of CNS infections in patients presenting to our hospital. The disease outcomes and follow-ups have not been reported. The final diagnosis in a few patients varied during their treatment. Nonetheless, in all these patients, CSF examination was clinically indicated to rule out CNS infections. Hence we examined these patients' CSF regardless of the final diagnosis if they matched the eligibility criteria and had a complete demographic, clinical and CSF lab data. Etiological diagnosis was established in 38% (n = 33) out of 87 analyzed. The diagnostic yield would have increased if only patients with the final diagnosis of meningitis/encephalitis were included in our analysis. As in many other studies, nearly two-third of the cases were undiagnosed. In 62% of our patients, the etiology was unidentified which may be due to other organisms especially *Mycobacterium tuberculosis* (TBM) which was not tested for in this study. The other possibilities may include: HIV, fungal, atypical organisms and other non infectious causes. The diagnostic yield of the tests conducted may have been affected due to prior treatment before hospital admission. The test results for PCR/serology may have been affected by an early/late presentation of some study patients to our hospital. Out of 87 studied, 14 CSF samples were insufficient for serological testing and 19 culture reports were unavailable which may have further lowered the total etiological diagnosis. Finally, the simultaneous molecular and serological tests conducted for different pathogens despite being very helpful in understanding the cause of CNS infections are certainly not cost-effective in our routine patient care.

Central nervous system (CNS) infections are a common cause of mortality and morbidity in Nepal. The identification of the pathogens responsible for CNS infections in Nepal has been difficult due to lack of modern diagnostic facilities, late presentation and widespread use of antibiotics prior to any hospital admission limiting the diagnostic yield of bacterial culture. The routine use of molecular diagnostics with a better diagnostic yield is very limited due to high costs. Our study attempts to identify CNS pathogens using molecular diagnostics and serology in adults admitted to a tertiary referral hospital in Kathmandu, Nepal. JEV and enteroviruses were the most common pathogens detected in adults with a CNS infection in our setting. Although previous studies have reported JEV as an important CNS pathogen, our study shows that enteroviruses too are a common cause of CNS infections in Nepal.

## Methods

We conducted a prospective observational study to identify the aetiology of CNS infections in Kathmandu, Nepal. The study site was Patan hospital: a 450 bed tertiary care referral centre with 320,000 outpatients and 20,000 in patients annually.

All patients admitted in the Emergency/Medical ward between February 2009 to April 2011 were eligible if they met the following inclusion criteria: >14 years of age; at least one of the following symptoms or signs: fever, headache, vomiting, neck stiffness, altered consciousness and having a cerebrospinal fluid (CSF) sample taken. Patient not meeting the above mentioned criteria or having an incomplete demographic/clinical/CSF lab data (glucose, protein, WBC, differential count) were not included in the analysis.

The study was approved by Patan Hospital Ethical Committee. All enrollees signed an informed consent form prior to enrolment. In those less than 16 years of age and in unconscious patients, a parent or guardian was asked to provide a written informed consent.

Demographic and clinical data, data from routine hematology and biochemistry analysis of blood and CSF (glucose, protein, WBC, differential count) were collected and recorded on case record forms. CSF, plasma and serum samples for aetiological analysis were obtained at enrolment. Samples were stored at −80°C and later transferred to the Oxford University Clinical Research Unit (OUCRU) in Ho Chi Minh City, Vietnam. Tests for Human immunodeficiency virus and tuberculosis infections were not performed during our analysis but were done during their hospital stay if clinically indicated.

### Microbiological Investigations

Blood and CSF cultures were performed in the microbiology laboratory of Patan hospital. Blood culture was performed in media containing tryptic soy broth and sodium polyanethole sulphonate; incubated at 37°C and examined daily for 7 days. CSF samples were Gram stained and plated on MacConkey and blood agar plates and incubated for 24 hours.

### Molecular diagnostic analyses

Nucleic acids were extracted from CSF specimens using the Roche MagnaPure MP96 (Roche, Mannheim, Germany), and subsequently analyzed for bacterial infections using internally controlled real-time PCR for detection of 4 bacterial pathogens that are most frequently associated with meningitis in Southeast Asia: *S. pneumoniae, H. influenzae* type b (Hib), *N. meningitidis*, and *S. suis*. Real-time (RT-) PCRs were also used to detect Herpes Simplex Virus (HSV), Varicella Zoster Virus (VZV), enteroviruses (generic and EV71), Human parechovirus, and Nipah Virus^39^. The nucleic acid extraction protocols, primers and probe sequences and thermocycling protocols used were as described in previous studies done in Vietnam^40^.

### Serology

A capture IgM ELISA (Venture technologies, Sarawak, Malaysia) that utilizes inactivated antigens from JEV and Dengue virus (DENV) was used to detect JEV specific IgM antibodies in CSF specimens, and to distinguish them from (cross-reactive) DENV specific IgM antibodies.

### Statistical Analysis

Demographic, clinical, CSF and hematology data of the total patients were categorized into bacterial (*N.meningitidis, S.pneumoniae, S.aureus*), viral (enteroviruses, JEV, HSV) and dual infections (bacterial + viral or viral + viral) based on (RT-) PCR, culture and serology results [Table 1 and Table 3]. The categorical variables were presented as number (percentage) and the continuous variables were presented as median (Inter Quartile Range-IQR). Categorical variables were compared using Fisher's exact test and Mann- Whitney U test was used to compare continuous variables [Table 3]. A two-tailed p value <0.05 was considered to indicate statistical significance.

## Author Contributions

A.A., S.K., B.B. and J.F. designed the study. R.V.D., L.V.T. and B.N.T.T.C. conducted the experiment. R.S., O.S., S.D.T., A.G., R.K.K., K.R.P. and M.S. collected the data. A.G., R.K.K., S.D.T. analyzed the data. A.G., R.V.D. and B.B. wrote the manuscript with additional inputs from L.V.T., B.N.T.T.C., J.F., A.A., S.K., A.K., S.D. and R.K.K. All the authors reviewed and approved the manuscript.

## Figures and Tables

**Table 1:** Demographic, clinical and lab data of patients diagnosed with PCR, serology and culture as bacterial meningitis or aseptic meningitis/encephalitis

	Total enrollees	Enteroviruses	JEV	*N.meningitidis*	S.pneumoniae*	HSVΦ	*S.aureus*#	Dual infections
	N = 87	N = 9	N = 7	N = 5	N = 3	N = 2	N = 1	N = 5
Age	30(22–54)	39(33–53)	42(27–67)	24(20–30)	22(22–45)	21(35)	27	24(22–27)
Male	50(57)	5(55)	4(57)	4(80)	2(66)	1(50)	1	3(60)
Median duration of illness	7(8–14)	7(6–14)	7(4–7.5)	2(1.5–2)	9(1–30)	7(90)	45	10(3–30)
No previous treatment/referral	39(44)	5(55)	2(28)	4(80)	2(66)	1(50)	1	1(20)
Clinical Features								
Fever	77(89)	8(88)	7(100)	5(100)	3(100)	2(100)	2	5(100)
Headache	56(64)	5(55)	5(71)	4(80)	1(33)	1(50)	2	5(100)
Vomiting	52(60)	5(55)	5(71)	5(100)	1(33)	1(50)	1	3(60)
Alteration of consciousness	37(42)	4(44)	4(57)	1(20)	1(33)	1(50)	0	2(40)
Seizure	16(18)	1(11)	1(14)	1(20)	1(33)	0	0	1(20)
Neck stiffness	48(55)	5(55)	3(42)	4(100)	3(100)	1(50)	1	4(80)
GCS<15	35(40)	4(44)	3(42)	1(20)	1(33)	0	0	1(20)
Travel	10(11)	1(11)	1(14)	2(40)	0	0	0	0
CSF								
Glucose(g/dl)	53(31–67.5)	53(37–56)	79(47.5–95.5)	18(5–38)	12(10–26)	55(43)	70	42(29–58)
Protein(g/dl)	90(30–185.5)	78(29–218)	144(135–162)	300(266–330)	330(168–354)	54(137)	39	82(48–152)
WBC(10e6/ml)	22(5.5–167.5)	25(2–64)	42(25.5–111.5)	630(480–1500)	140(109–1120)	30(18)	3	180(140–1120)
Neutrophils (%)	10(1–40)	15(1–20)	10(7–25)	85(80–90)	84(10–96)	70(5)	1	47(25–70)
Lymphocytes (%)	12(3–70)	72(1–80)	70(30–85)	10(10–15)	16(4–90)	30(95)	2	53(30–75)
Hematology								
TLC(10e6/ml)	10.7(8.3–14.65)	6.7(6.5–10.1)	9.1(8.75–14)	15(10.7–21.1)	8.7(18.1–27.1)	13.2(5)	5.8	10.3(10.1–14.9)
Neutrophils (%)	80(70–89.5)	85(66–90)	86 (73–89)	95(89–97)	86(84–95)	70(40)	63	90(80–92)
Lymphocytes (%)	19.5(10–29)	15(10–30)	14(11–27)	5(3–11)	14(5–16)	28(55)	34	8(7–16)

^1^Values represent number (percentage) in categorical data and median (Inter-quartile range; IQR) in continuous data in total enrollees, JEV, enteroviruses, *N. meningitidis*, and dual infections.

^2^*Data for *S. pneumoniae* -values represent median (percentage) in categorical data and median (range) in continuous data.

^3^ΦData for 2 patients with HSV infection are tabulated with one in the bracket ( ). Categorical data are presented as number (percentage) and individual patient data is tabulated for both patients [with one in bracket ( )] in categorical variables.

^4^#Individual patient's data is tabulated for *S. aureus*.

^5^Dual infections (n = 5) = bacterial + viral (n = 4); viral + viral (n = 1).

**Table 2:** Laboratory confirmed etiology of CNS infections for patients enrolled in the study

Pathogens	PCR	Serology	Culture
Bacteria			
*S. pneumoniae*	3(3.4)	_	0(0)
*N. meningitidis*	5(5.7)	_	0(0)
*S. suis*	0(0)	_	0(0)
*H. influenzae*	0(0)	_	0(0)
*S.aureus*	_	_	1(1.4)
Viruses			
Enteroviruses(EVs)	9(10.3)	_	_
EV71	0(0)	_	_
JEV	_	7(9.5)	_
Equivocal JEV	_	1(1.3)	_
Dengue virus	_	0(0)	_
Herpes Simplex Virus	2(2.2)	_	_
Varicella zoster Virus	0(0)	_	_
Human parechovirus	0(0)	_	_
Nipah virus	0(0)	_	_
Dual infections			
*N. meningitidis* + JEV	1(1.1)	1(1.3)	0(0)
*S. pneumoniae* + JEV	1(1.1)	1(1.3)	0(0)
Enteroviruses + JEV	1(1.1)	1(1.3)	0(0)
Enteroviruses + *S. pneumoniae*	2 [1 + 1](2.2)*	_	0(0)
Enteroviruses + *S. aureus*	1(1.1)	_	1(1.4)
Total positive	25(28.7)	11(15)	2(2.8)
Total sample for analysis	87	73	68

The total number of the laboratory confirmed etiology in numbers and percentage in brackets ( ).

[-] tests not done for a particular pathogen.

*2 (RT) PCR positive results for enteroviruses and S.pneumoniae in same patient.

**Table 3:** Comparison of patients' characteristics grouped as bacterial, viral and dual CNS infections

	Bacterial*	Viral$		Viral	Dual #		JEV	Enteroviruses		Bacterial	Dual	
	N = 9	n = 18	p value	N = 18	N = 4	p value	N = 7	N = 9	pvalue	N = 9	N = 4	pvalue
Age	24(22–30)	38(29–63)	0.02	38(29–63)	23(21.25–24.75)	0.02	42(27–67)	39(33–53)	0.87	24(22–30)	23(21.25–24.75)	0.58
Male	6(67)	10(55)	0.69	10(55)	2(50)	1	4(57)	5(55)	1	6(67)	2(50)	1
Median duration of illness	2(1–9)	7(4.5–12.5)	0.15	7(4.5–12.5)	16.5(3–45)	0.86	7(4–7.5)	7(6–14)	0.59	2(1–9)	16.5(3–45)	0.24
No previous treatment/referral	5(55)	8(44)	0.69	8(44)	1(25)	0.06	2(28)	5(55)	0.35	5(55)	1(25)	0.55
Clinical Features												
Fever	9(100)	17(94)	1.00	17(94)	4(100)	1.00	7(100)	8(88)	1	9(100)	4(100)	1
Headache	7(77)	11(61)	0.66	11(61)	4(100)	0.26	5(71)	5(55)	0.63	7(77)	4(100)	1
Vomiting	7(77)	11(61)	0.66	11(61)	3(75)	1.00	5(71)	5(55)	0.63	7(77)	3(75)	1
Alteration of consciousness	2(22)	9(50)	0.22	9(50)	1(25)	0.59	4(57)	4(44)	1	2(22)	1(25)	1
Seizure	2(22)	2(10)	0.58	2(10)	1(25)	0.47	1(14)	1(11)	1	2(22)	1(25)	1
Neck stiffness	8(88)	9(50)	0.09	9(50)	3(75)	0.59	3(42)	5(55)	1	8(88)	3(75)	1
GCS<15	2(22)	7(38)	0.66	7(38)	0(0)	0.26	3(42)	4(44)	1	2(22)	0(0)	1
Travel	3(33)	2(11)	0.29	2(11)	0(0)	1.00	1(14)	1(11)	1	3(33)	0(0)	0.49
CSF												
Glucose(g/dl)	18(10–38)	55(39.25–75.75)	0.02	55(39.25–75.75	35.5(24.25–49.5)	0.25	79(47.5–95.5)	53(37–56)	0.10	18(10–38)	35.5(24.25–49.5)	0.31
Protein(g/dl)	300(168–330)	134.5(60–166)	0.03	134.5(60–166)	117(73–189)	0.83	144(135–162)	78(29–218)	0.18	300(168–330)	117(73–189)	0.28
WBC(10e6/ml)	630(24–2500)	31.5(12–62.25)	0.03	31.5(12–62.25)	630(133.25–1285)	0.01	42(25.5–111.5)	25(2–64)	0.56	630(24–2500)	630(133.25–1285)	0.87
Neutrophils (%)	89(84–95)	12.5(3.25–26)	0.01	12.5(3.25–26)	58.5(41.5–74.5)	0.01	10(7–25)	15(1–20)	0.49	89(84–95)	58.5(41.5–74.5)	0.64
Lymphocytes (%)	10(10–16)	65(5.25–80)	0.42	65(5.25–80)	41.5(25.5–58.5)	0.76	70(30–85)	72(1–80)	0.22	10(10–16)	41.5(25.5–58.5)	0.21
Hematology												
TLC(10e6/ml)	15(10–21.1)	8.75(6.52–12.9)	0.03	8.75(6.52–12.9)	12.5(9.65–17.25)	0.18	9.1(8.75–14)	6.7(6.5–10.1)	0.31	15(10–21.1)	12.5(9.65–17.25)	0.64
Neutrophils (%)	89(84–95)	82(66–89)	0.07	82(66–89)	91(86.75–92.25)	0.10	86 (73–89)	85(66–90)	0.67	89(84–95)	91(86.75–92.25)	0.75
Lymphocytes (%)	11(5–16)	16(10.75–33)	0.09	16(10.75–33)	7.5(6.5–11.75)	0.07	14(11–27)	15(10–30)	0.87	11(5–16)	7.5(6.5–11.75)	0.87

*Bacteria- *N. meningitidis* (n) = 5, *S.pneumoniae* (n) = 3, *S aureus* (n) = 1.

$Viruses- enteroviruses (n) = 9, JEV (n) = 7, HSV (n) = 2.

#Dual – Bacterial + viral infections (n) = 4. Bacterial/viral infections were compared with dual infections (bacterial + viral co-infection); co-infection with 2 viruses (virus + virus) [n = 1] was not included in dual infections in the comparative analysis.

Values represent number (percentage) in categorical data and median (Inter-quartile range; IQR) in continuous data.

Fisher's exact test was used for categorical variables and Mann-Whitney U test was used for continuous data to compare-- bacterial vs. viral infections; viral vs. dual infections; enteroviruses vs. JEV and bacterial vs. dual infections. Two tailed tests p value were considered to detect any statistical significance.
